# Short‐chain fatty acids enhance muscle mass and function through the activation of mTOR signalling pathways in sarcopenic mice

**DOI:** 10.1002/jcsm.13573

**Published:** 2024-10-31

**Authors:** Chaoran Liu, Pui Yan Wong, Qianjin Wang, Hei Yuet Wong, Tao Huang, Can Cui, Ning Zhang, Wing Hoi Cheung, Ronald Man Yeung Wong

**Affiliations:** ^1^ Department of Orthopaedics & Traumatology The Chinese University of Hong Kong Hong Kong SAR China

**Keywords:** Aging, Gut microbiota, Muscle, Sarcopenia, Short‐chain fatty acids

## Abstract

**Background:**

Sarcopenia is a prevalent muscle disorder in old people leading to higher fracture rate, mortality, and other adverse clinical outcomes. Evidence indicates that short‐chain fatty acids (SCFAs), which are beneficial gut microbial metabolites, were reduced in old people with sarcopenia. This study aimed to determine whether the use of SCFAs as a supplement can be a therapeutic strategy of sarcopenia in a pre‐clinical model.

**Methods:**

Seven‐month‐old pre‐sarcopenic senescent accelerated mouse prone 8 (SAMP8) mice received daily SCFAs cocktail (acetate, butyrate, and propionate) for 3 months. Age‐matched senescence accelerated mouse resistant 1 (SAMR1) and SAMP8 mice receiving sodium‐matched drinking water were control groups. The gut microbiota composition analysis of aged mice with or without sarcopenia was conducted by 16S rDNA sequencing. Gut barrier‐related proteins and lipopolysaccharide (LPS) concentration were biomarkers of gut permeability. Colon inflammation levels, circulatory SCFAs concentration, muscle quality, function, and underlying pathways were detected by cell number counting, RT‐qPCR, gas chromatography–mass spectrometry, measurements of muscle wet weight and grip strength, ex vivo functional test, treadmill endurance test, transcriptomic sequencing, morphological and immunofluorescent staining, as well as western blot. To investigate the role of mTOR signalling pathways in SCFAs treatment, C2C12 myotubes were treated with rapamycin.

**Results:**

Aged SAMP8 mice had different microbiota composition, and lower serum butyric acid compared with SAMR1 mice (*P* < 0.05). SCFAs treatment reversed the increment of colon inflammation (2.8‐fold lower of *il‐1β*) and gut barrier permeability (1.7‐fold lower of LPS) in SAMP8 mice. Increased muscle mass, myofibre cross‐sectional area, grip strength, twitch and tetanic force were found in SCFAs‐treated mice compared with control SAMP8 mice (*P* < 0.05). Anti‐fatigue capacity (1.6‐fold) and muscle glycogen (2‐fold) also improved after SCFAs treatment (*P* < 0.05). Transcriptomic analysis showed that AMPK, insulin, and mTOR pathways were involved in SCFAs treatment (*P* < 0.05). Regulation of AKT/mTOR/S6K1 and AMPK/PGC1α pathways were found. SCFAs attenuated fat infiltration and improved mitochondria biogenesis of atrophic muscle. In vitro studies indicated that SCFAs inhibited FoxO3a/Atrogin1 and activated mTOR pathways to improve myotube growth (*P* < 0.05), and rapamycin attenuated the effect of SCFAs through the inhibition of mTOR pathways.

**Conclusions:**

This study demonstrated that bacterial metabolites SCFAs could attenuate age‐related muscle loss and dysfunction, and protein synthesis‐related mTOR signalling pathways were involved both in vivo and in vitro.

## Introduction

Sarcopenia is an age‐related muscle disorder, characterized by loss of muscle mass, strength, and poor physical performance. Older persons who have lower muscle mass with either reduced grip strength or physical functions are diagnosed with sarcopenia recommended by the European Working Group on Sarcopenia in Older People 2 (EWGSOP2) and Asian Working Group for Sarcopenia 2019 (AWGS2019).^1^ The global prevalence of sarcopenia ranges from 10% to 27% in people over 60 years,^2^ and reaches 40% in people older than 65 years.^3^ People with sarcopenia have higher risks of fall, fracture, hospitalization, poor clinical outcomes, and all‐cause mortality.^4^, ^5^, ^6^ As of now, treatments for sarcopenia are limited, in which only nutrition and resistance exercise training are clinically effective.^7^ There is currently no Food and Drug Administration (FDA)‐approved drugs for sarcopenia, and therefore, novel therapies are still warranted to reduce risks of sarcopenia related adverse clinical outcomes.

The well‐known pathogenic factors of sarcopenia include the imbalance between protein synthesis and degradation, mitochondrial dysfunction, low growth hormone, chronic inflammation, and increased intramuscular fat infiltration.^8^ Old frail people have gut microbiota dysbiosis, such as reduced *Faecalibacterium* and *Lactobacillus*, which is postulated as a novel pathogenesis of sarcopenia.^9^ The beneficial gut bacteria produce the main metabolites short‐chain fatty acids (SCFAs), of which acetate, propionate, and butyrate are most abundant.^10^, ^11^ A recent study also demonstrated that butyrate and acetate levels are decreased in people with sarcopenia,^12^ and the supplement of SCFAs may attenuate the disease. A lack of beneficial gut microbiota and metabolites further leads to gut barrier dysfunction, allowing pro‐inflammatory metabolite lipopolysaccharide (LPS) able to translocate to the circulatory system and harm remote organs, including muscle.^13^ SCFAs has been shown to played beneficial roles in muscle via regulations of lipid, glucose, and energy metabolisms.^14^

Butyrate can prevent diabetic nephropathy‐induced muscle atrophy through activating the PI3K/AKT/mTOR signalling pathways in *db/db* mice,^15^ and also improve muscle mass and mitochondrial function, but not muscle strength in aged mice.^16^ Long‐term use of acetic acid decreased expression of atrophy‐related genes (Atrogin‐1 and MuRF1), activated AMPK, and increased mitochondria biogenesis in aged rats.^17^ The SCFAs cocktail (acetate, propionate, and butyrate) that simulates physiological concentration can increase both muscle mass and function in germ‐free mice, which shows potential in preventing against sarcopenia.^18^ SCFAs can also benefit intestine health. Diet‐induced propionate and butyrate protect intestinal permeability integrity and decreased plasma LPS binding protein in women with intestinal barrier impairment.^19^ Therefore, the supplement of acetate and butyrate that are reduced in old people with sarcopenia, accompanied with propionate may improve the gut barrier and bring benefits in aging muscle. Although the single use of acetate or butyrate to treat muscle has been studied in various animal models, the combination of main SCFAs and the underlying molecular mechanisms in sarcopenia have not been investigated. In this study, we aimed to (i) investigate the effect of SCFAs cocktail on sarcopenic mice and C2C12 myotubes, and (ii) explore the underlying mechanisms in how SCFAs enhance muscle and intestine health.

## Material and methods

### Animal

Male senescent accelerated mouse prone 8 (SAMP8) and senescence accelerated mouse resistant 1 (SAMR1) were used from the Laboratory Animal Service Center (LASEC), and housed at the Experimental Animal Center at the Prince of Wales Hospital in Hong Kong under a 12‐h light/dark cycle, ambient temperature of 18–23°C, and 70% humidity, with food (LabDiet PicoLab Select Mouse 50 IF/9F) and water ad libitum. The background of these two lines of mice are AKR/J mice, and SAMP8 has accelerated aging phenotypes, especially in muscle.^20^ Male mice were used since their hormone fluctuation was relatively less.^21^ 10‐month sarcopenic SAMP8 mice (P8) and SAMR1 mice (R1) without any treatment were used for stool sample collection (*n* = 8 per group). The study was approved by the Animal Experimentation Ethics Committee of The Chinese University of Hong Kong (Ref: 20‐284‐MIS).

### SCFAs treatment

SAMP8 mice were randomized into control (CTL P8), SCFAs treatment (SCFAs P8) groups, and SAMR1 mice (CTL R1) at 7 months of age as non‐sarcopenic control group. SCFAs‐treated mice received daily SCFAs (sodium acetate 67.5 mM, sodium butyrate 40 mM, sodium propionate 25.9 mM) (Sigma Aldrich, USA) in drinking water at their 7 months old for 3 months, as muscle conditions of P8 rapidly declined after 7 months old.^20^ Age‐matched R1 and P8 mice were administrated with equal sodium water as control groups (*n* = 8 per group). The drinking volume of control and SCFAs groups was recorded. As P8 had significant reduction of muscle mass and function at 10 months old,^20^ all mice were sacrificed at this age. Mice were euthanized by ketamine and xylazine without fasting.

### Stool sample DNA extraction and 16S rDNA sequencing

Mice were placed into a sterile box individually for fresh stool collection in the morning. Details of sequencing method seen in Supporting Information.

### Gas chromatography–mass spectrometry analysis of serum SCFAs

Blood samples were drawn from mice and centrifuged at 4000 rpm for 10 min. Details of gas chromatography‐mass spectrometry (GC–MS) analysis method seen in Supporting Information.

### Serum ELISA analysis of LPS

Enzyme linked immunosorbent assay (ELISA) kits (CSB‐E13066m, CUSABIO, USA) were used to detect the levels of LPS in serum samples. Briefly, serum samples were diluted to an optimal concentration, followed by incubation, washing, and determination of absorbance based on the manufacturer's instructions.

### Muscle mass and function assessment

Wet muscle weight of lower limbs including tibialis anterior (TA), extensor digitorum longus (EDL), soleus (SOL), quadriceps (QUA), and gastrocnemius (GAS) was measured. Forepaw grip strength test (Mark‐10 Corporation, NY, USA) was conducted before sacrifice. Fresh GAS muscle was isolated from mice under anaesthesia for ex vivo functional test (800A, Aurora Scientific, Aurora, ON, Canada) as previously reported.^22^ The running endurance test was performed on the treadmill (76‐0895, Panlab, Harvard Apparatus, MA, USA) 1 week before sacrifice following previous protocols.^23^ Briefly, mice received treadmill training (10 min at 17 cm/s, slope at 10°) three times before the formal testing. Mice were then exposed to a protocol consisting of 10 min at 10 cm/s, followed by 40 min at 29 cm/s. After that, the speed was gradually increased by 1 cm/s until mice were exhausted defined as falling back to the grid 3 times within 30 s.

### Muscle and liver tissue glycogen quantification

Glycogen contents of QUA muscle and liver tissue in mice were quantified with Glycogen Assay Kit II (ab169558, Abcam, Cambridge, UK) following the manufacturer's instructions.

### Colon, muscle, and heart section preparation and staining

Samples of colon (paraffin), gastrocnemius (frozen), and heart (paraffin) were sectioned into 4 μm, 8 μm, and 4 μm, respectively. Colonic H&E staining was performed. The neutrophils and eosinophils number in the mucosa of each image was then counted and recorded with Image J software (NIH, MD, USA). Immunofluorescent staining of colon using anti‐Mucin 2 (Muc2) (1:200; ab272692, abcam, UK), anti‐Claudin1 (1:200; ab15098, abcam, UK), and anti‐Occludin antibodies (1:200; A2601, Abclonal, USA) overnight at 4°C, as well as secondary antibodies goat anti‐rabbit Alexa Fluor® 488 or 594 (1:500; Thermo scientific, USA) for 1 h at room temperature was performed to determine the location of proteins. Muscle H&E staining, and myosin heavy chain (MHC) staining using anti‐MHC I (BA‐F8; 9.3:100), anti‐MHC IIa (SC‐71; 6.2:100), anti‐MHC IIb (BF‐F3; 7.7:100) (DSHB, University of Iowa, USA), and Alexa Fluor® 350 IgG_2b_, Alexa Fluor® 488 IgG_1_, Alexa Fluor® 555 IgM (1:500; Thermo scientific, USA) were conducted for myofibre cross‐sectional area (CSA) and twitch type detection. Oil red O staining of muscle section was also executed and analysed as previous protocols (Figure S1).^24^ Sirus red and H&E staining was performed to identify the influence of sodium drinking water on the mouse heart. The image was captured under microscope (Leica Microsystems Ltd., Germany).

### Transcriptome sequencing (RNA‐seq) of muscle tissue

Total RNA was extracted from TA muscle of CTL R1, CTL P8, SCFAs P8 mice (*n* = 8 per group) using Trizol reagent (Invitrogen, CA, USA) according to the the manufacturer's guidelines. Two rounds of purification were performed using Dynabeads Oligo (dT) (Thermo Scientific, CA, USA) for purifying mRNA from total RNA. Purified mRNA was fragmented under 94°C for 5–7 min using the Magnesium RNA Fragmentation Module (e6150, NEB, USA). cDNA was reverse‐transcribed from cleaved RNA fragments and used to establish the cDNA library. The transcriptome sequencing was performed on the Illumina Novaseq™ 6000 platform (Illumina, Inc., CA, USA). Reads were filtered and aligned to the corresponding genome using Cutadapt and HISAT2 package. Gene abundance quantification based on FPKM was analysed by StringTie, gffcompare, and ballgown.

### C2C12 culture and proliferation with SCFAs at different concentrations

C2C12 myoblasts were cultured in high‐glucose Dulbecco's Modified Eagle's medium (HG‐DMEM) containing 10% fetal bovine serum (FBS). 60:25:15 (acetate, propionate, and butyrate) was used as the ratio of SCFAs to treat C2C12.^18^ Based on the ratio, 0.6 mM, 0.25 mM, 0.15 mM were used as the standard concentration (as 1). The half as 0.5‐, and 2‐, 3‐, and 4‐fold concentrations of SCFAs cocktail were also used to detect the optimal interventional concentration by utilizing MTT Assay Kit (ab211091, Abcam, Cambridge, UK) following the instructions. 10^4^ mL^−1^ myoblasts were seeded.

### C2C12 differentiation with SCFAs, LPS, or rapamycin

C2C12 cell lines were differentiated from myoblasts to myotubes in HG‐DMEM with 2% non‐heat‐inactivated (HI) horse serum. LPS, which can be secreted by gut microbiota, is associated with gut barrier function.^25^ To mimic the in vivo environment, a lower dose of LPS was used, which is different from critical illness/sepsis of higher dose.^26^ 100 ng/mL LPS (Sigma Aldrich, USA) was added with or without SCFAs cocktail (0.6, 0.25, and 0.15 mM) at differentiation day 5 to mimic the systemic environment of sarcopenic mice after SCFAs treatment.^15^ Untreated (CTL), SCFAs‐treated (SCFAs), LPS‐treated (LPS), LPS, and SCFAs treated (LPS + SCFAs) groups were investigated. After 24 h, myotubes of these groups were harvested. Similarly, to investigate whether the role of SCFAs on myotubes is mediated through mTOR signalling pathways, 60 nM rapamycin (HY‐10219, MCE, USA) as an inhibitor of mTOR complex 1 (mTORC1) was added into C2C12 myotubes at day 3. At day 5, SCFAs were added into the control and rapamycin‐treated groups. Four groups CTL, SCFAs, rapamycin (Rapa), rapamycin with SCFAs (Rapa + SCFAs) were investigated. After 24 h, cells were harvested for protein extraction. Rapamycin has been added for a total 96 h. C2C12 myotubes under the LPS condition were also detected and grouped as LPS, LPS + SCFAs, LPS + Rapa, and LPS + Rapa + SCFAs groups. Cell studies were repeated at least twice with three to six replicates each time.

### C2C12 myotube staining

C2C12 myotubes were cultured and treated as above. After the intervention, cells were washed, fixed, and stained by MHC IIa antibody (1:100; SC‐71) overnight at 4°C and Alexa Fluor® 488 IgG_1_ (1:500) for 1 h at room temperature. The myotube diameter length and number of nuclei were measured based on the image.

### RNA extraction and real‐time PCR

RNA from EDL muscle, colon, and C2C12 myotubes were extracted. Details seen in Supporting Information.

### Western blot

Samples of TA, colon, and C2C12 myotubes were used. Details of method and antibodies seen in Supporting Information.

### Statistical analysis

Data were shown as mean ± standard deviation (SD), or quartiles. Student's unpaired *t*‐test, one‐way ANOVA with post‐hoc analysis, or two‐way ANOVA with Šídák's multiple comparison test were performed. In 16S rDNA sequencing analysis, Chao1, Shannon, and Simpson as alpha diversity, and Bray–Curtis distance‐based PCoA as beta diversity were shown and its significance was performed by permutational multivariate ANOVA (PERMANOVA). Alpha diversity refers to microbe richness within a community, and beta diversity refers to microbe diversity between communities. Linear discriminant analysis effect size (LEfSe) is an algorithm for high‐dimensional data, such as microbial components. Linear discriminant analysis (LDA) score > 4 was the cut‐off to detect differential gut microbiota. As for the transcriptomic sequencing, Benjamini and Hochberg false discovery rate (FDR) was calculated to adjust the *P* value and obtain the *q* value to identify differential genes between groups. Differential genes were clustered and annotated by KEGG pathways. SPSS (Version 20.0, SPSS Inc, IBM, USA), R (version 4.0.2), and Graphpad (Prism9, GraphPad Software, USA) were utilized. *P* or *q* value < 0.05 was regarded as significant difference.

## Results

### Gut microbiota composition in aged mice with sarcopenia, and SCFAs, LPS, and colonic cytokine changes after SCFAs treatment

**Figure 1:**
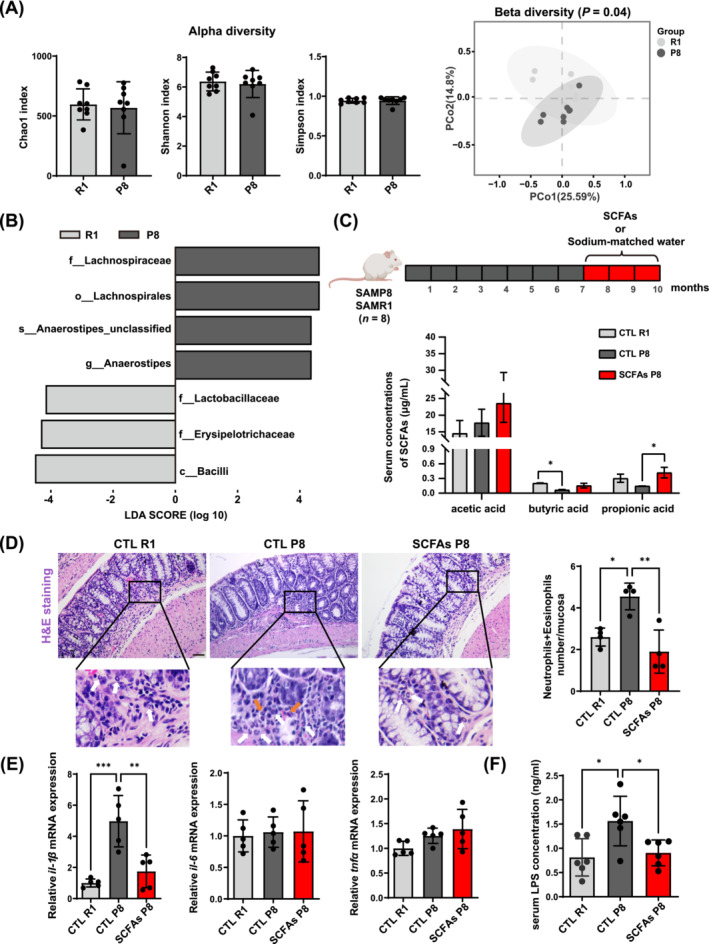
Gut microbiota characteristics between SAMR1 and SAMP8 mice, and serum indicators and inflammation levels after SCFAs treatment. (A) Alpha diversity and beta diversity (PCoA based on Bray–Curtis distance) between SAMR1 (R1) and SAMP8 (P8) (*n* = 8). (B) Lefse (LDA score > 4, indicating large degree of separation between groups with *P* < 0.05) of differential gut microbiota between P8 and R1 groups (*n* = 8). Higher abundance of microbes in R1 and P8 were shown in light grey and dark grey, respectively. Taxonomic rank: o, order; c, class; f, family; g, genus, s, species. (C) SCFAs or sodium‐matched water administration protocol in P8 and R1 mice. SCFAs concentration in serum between CTL R1, CTL P8, and SCFAs P8 mice (*n* = 3). (D) H&E staining and average numbers of colonic neutrophils (white arrow) and eosinophils (orange arrow) (*n* = 4 mice per group, five sites per mouse, scale bar = 50 μm). (E) mRNA expression of cytokines in colon (*n* = 5). *(F)* Serum LPS concentration amongst three groups (*n* = 6). * *P* < 0.05, ** *P* < 0.01, *** *P* < 0.001, by Student's unpaired *t*‐test, or one‐way ANOVA with Tukey's analysis.

Sarcopenic P8 and non‐sarcopenic R1 mice had different gut microbiota composition reflected in beta diversity, but similar alpha diversity was found (Figure 1A). Sarcopenic mice had higher abundance of family Lachnospiraceae, and its genus *Anaerostipes*, but lower abundance of family Erysipelotrichaceae and Lactobacillacea (Figure 1B). Non‐sarcopenic mice had higher serum concentration of butyric acid compared with sarcopenic mice, which had no difference to P8 mice after SCFAs treatment. Treated mice also had increased propionic acid compared with P8 mice (Figure 1C). More inflammatory cells (neutrophils and eosinophils) were found in P8 mice compared with other groups (Figure 1D). The mRNA expression of *il‐1β* in colon tissue increased in P8 mice, and was reduced after the treatment (Figure 1E). Increased serum LPS level in P8 mice could be reversed by SCFAs (Figure 1F). SCFAs and sodium‐matched drinking water did not significantly affect the heart structure compared with untreated mice (Figure S2A).

### Intestinal permeability‐related proteins improved after the SCFAs treatment

**Figure 2:**
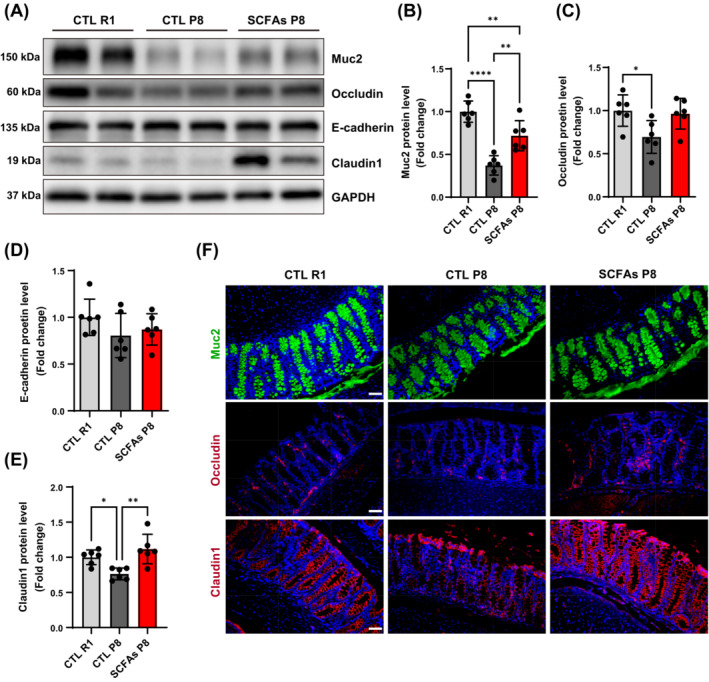
Levels and location of intestinal barrier protein. (A–E) Protein levels of Muc2, Occludin, E‐cadherin, and Claudin1 (*n* = 6). (F) Immunofluorescent staining of protein that significantly different, including Muc2, Occludin, and Claudin1 (*n* = 4 mice per group, scale bar = 50 μm). *P < 0.05, **P < 0.01, ****P < 0.0001, by one‐way ANOVA with Tukey's analysis.

P8 mice had lower colonic protein levels of Muc2 in goblet cells, and tight junction protein Occludin as well as Claudin1 compared with R1 mice signifying increased permeability. After SCFAs treatment, higher Muc2 and Claudin1 protein levels in the SCFAs‐treated P8 mice were found compared with control P8 mice, but E‐cadherin was comparable amongst groups (Figure 2A–E). The locations of differential proteins were shown by immunofluorescent staining (Figure 2F).

### Muscle phenotype change after SCFAs treatment

**Figure 3:**
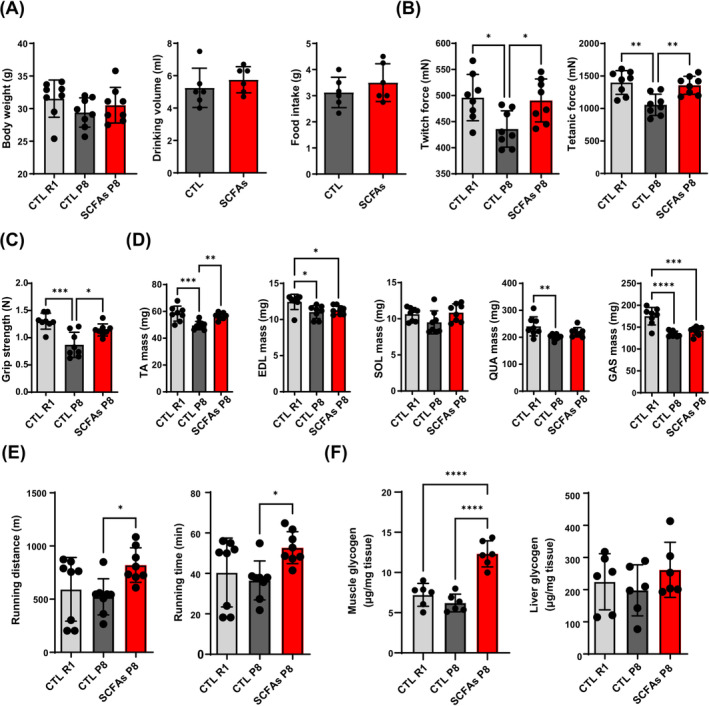
Muscle phenotypes of CTL R1, CTL P8, and SCFAs‐treated P8 mice. (A) Body weight amongst groups (*n* = 8). Daily drinking volume and food intake per mouse between sodium‐matched and SCFAs groups (*n* = 6, 2 mice per cage, 3 cages for two‐day measurement). (B, C) Functional tests of ex vivo GAS muscle function, and grip strength (*n* = 8). (D) Lower limb muscle mass (*n* = 8). (E) Running distance and time of endurance test (*n* = 8). (F) Glycogen contents in QUA muscle and liver (*n* = 6). *P < 0.05, **P < 0.01, ***P < 0.001, ****P < 0.0001, by Student's unpaired *t*‐test, or one‐way ANOVA with Tukey's analysis.

**Figure 4:**
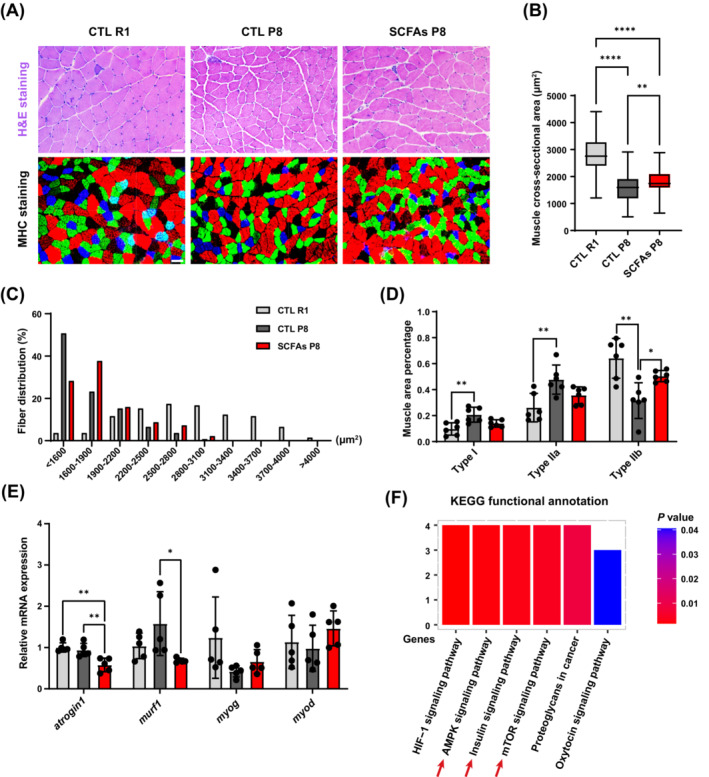
Myofibre characteristics and muscle transcriptome‐based functional annotation amongst groups. (A) GAS muscle H&E and MHC staining (*n* = 6 mice per group, scale bar = 50 μm). (B, C) Calculation of myofibre CSA (23 myofibres per mouse, *n* = 6 mice per group). (D) Calculation of fibre type proportion according to muscle area. (E) EDL muscle mRNA expression of myogenesis and atrophic genes amongst groups (*n* = 5). (F) TA muscle KEGG functional annotation of differential genes (*q* < 0.05) (*n* = 8). *P < 0.05, **P < 0.01, ****P < 0.0001, by Student's unpaired *t*‐test, or one‐way ANOVA with Tukey's analysis.

SCFAs treatment did not change the body weight or affect drinking volume and food intake of mice (Figure 3A). P8 mice had lower grip strength, GAS muscle twitch and tetanic force than R1 mice, which could be improved by SCFAs (Figure 3B,C). R1 mice had higher TA, EDL, QUA, and GAS mass than P8 mice. SCFAs‐treated P8 mice had significantly increased TA mass (Figure 3D). Additionally, treated P8 mice exhibited improved anti‐fatigue capacity as measured by treadmill endurance test (Figure 3E). More than 2‐fold muscle glycogen content was found in SCFA‐treated P8 mice compared with other groups, but the liver glycogen content was comparable (Figure 3F). R1 mice had the highest GAS myofibre CSA, and SCFAs‐treated P8 mice had higher CSA than control P8 mice (Figure 4A–C). MHC staining showed that control P8 mice had higher ratio of type I (slow) and type IIa fibre (fast oxidative), but lower type IIb (fast) fibre in GAS muscle. SCFAs only increased type IIb muscle fibre area proportion (Figure 4A,D). The expression of myogenesis genes (*myod*, *myog*) was similar, but reduced atrophic genes (*atrogin1* and *murf1*) expression in EDL muscle was found in the SCFAs‐treated compared with control P8 mice (Figure 4E).

### The effect of SCFAs on insulin‐like growth factor (IGF) system and mTOR signalling pathways in muscle

**Figure 5:**
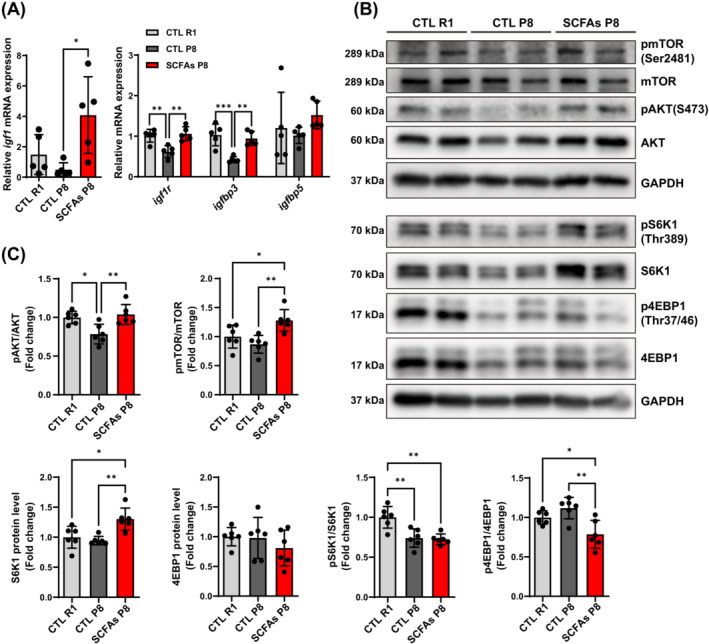
Effects of SCFAs on insulin and mTOR signalling pathways. (A) EDL muscle mRNA expression of genes related to IGF system (*n* = 5). (B, C) Activation of AKT/mTOR signalling pathways and expression of downstream proteins in TA muscle (*n* = 6). *P < 0.05, **P < 0.01, ***P < 0.001, by one‐way ANOVA with Tukey's analysis.

The RNA‐seq result of TA muscle showed the up‐regulation of 49 genes, and down‐regulation of 17 genes in SCFA‐treated mice compared with control P8 mice (*q* < 0.05). We performed KEGG annotation by using differential genes (*q* < 0.05) and found HIF‐1 (*pfkfb3*, *mknk2*, *mtor*, and *hk2*), AMPK (*pfkfb3*, *cab39*, *prkag2*, and *mtor*), insulin (*mknk2*, *prkag2*, *mtor*, and *hk2*), and mTOR (*clip1*, *cab39*, *lpin1*, and *mtor*) signalling pathways were mostly clustered (*P* < 0.05). Besides, the oxytocin pathway (*rcan1*, *prkag2*, and *nfatc1*) was also involved, in which genes played roles in muscle fibre types (Figure 4F). Other pathways that did not show significance were not well clustered (*P* > 0.05). However, chemokine signalling pathway and cytokine‐cytokine receptor interaction, which were mainly regarded as transcriptome differences between R1 and P8, were not reversed by SCFAs. We first detected mRNA expression of IGF system‐related genes of muscle, and found higher *igf1*, IGF1 receptor (*igf1r*), IGF binding protein 3 (*igfbp3*) in EDL muscle of SCFA‐treated P8 mice than control P8 mice, whilst R1 mice had comparable *igf1*, *igf1r*, and *igfbp3* levels compared with treated P8 mice (Figure 5A). mTOR signalling pathways are important in muscle protein synthesis. Herein, we investigated the activation of AKT/mTOR pathways in TA muscle. AKT/mTOR phosphorylation levels were significantly increased in SCFAs‐treated compared with control P8 mice. SCFAs‐treated P8 mice had highest protein levels of mTOR downstream S6K1, and lower phosphorylation of 4EBP1 levels compared with control P8 mice (Figure 5B,C), whilst R1 mice had the highest activation of S6K1 and higher pS6K1 than control P8 mice (Figure S2B).

### AMPK pathway activation and intramuscular fat changes after the treatment

**Figure 6:**
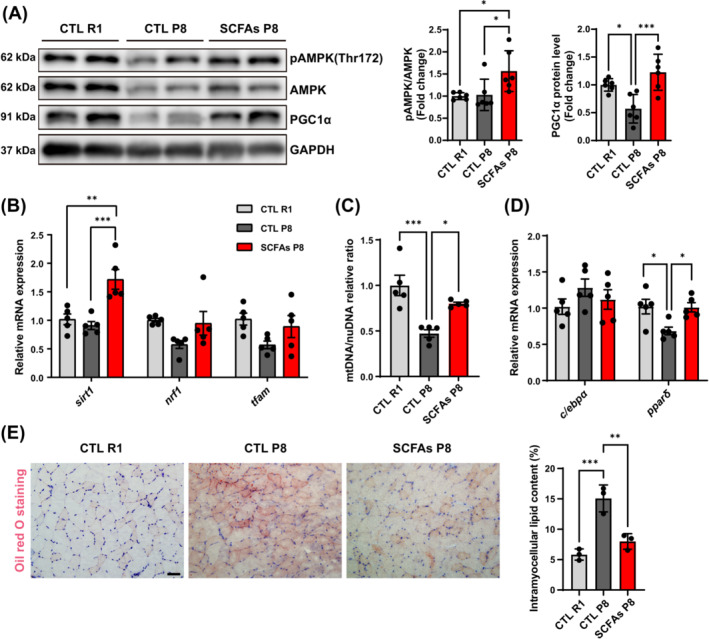
AMPK activation, mRNA expression of mitochondria‐related genes, muscle fat and lipid metabolism genes expression. (A) AMPK activation and PGC1α at protein level in TA muscle (*n* = 6). (B, C) EDL muscle mRNA expression of genes related to mitochondria biogenesis, and ratio of mtDNA/nuDNA (*n* = 5). (D) EDL muscle mRNA expression of lipid metabolism genes (*n* = 5). (E) Average intramuscular lipid content of GAS muscle (*n* = 3 mice per group, seven sites per mouse, scale bar = 50 μm). *P < 0.05, **P < 0.01, ***P < 0.001, by one‐way ANOVA with Tukey's analysis.

AMPK activation was found in TA muscle of SCFAs‐treated P8 mice. The up‐regulated *ppargc1a* in treated P8 mice was found in RNA‐seq (log_2_FC = 1.03, *q* = 0.053) compared with control mice. Similarly, control P8 mice had the lowest PGC1α protein level compared with other groups (Figure 6A). The mRNA expression of mitochondria biogenesis‐related genes (*sirt1*, *nrf1*, and *tfam*) was also detected, but only *sirt1* significantly improved in the treated group (Figure 6B). The transcriptional mtDNA copy number also increased in SCFAs‐treated P8 mice compared with control P8 mice (Figure 6C). It is known that AMPK signalling pathways are also related to fatty acid oxidation. The mRNA expression of *ppar*δ increased after the treatment, but *c/ebpα* was similar (Figure 6D). The staining of lipid in the GAS muscle showed increased intramuscular fat in control P8 mice, which was decreased after SCFAs treatment (Figure 6E).

### C2C12 myoblast proliferation and roles of SCFAs on myotubes with or without LPS

**Figure 7:**
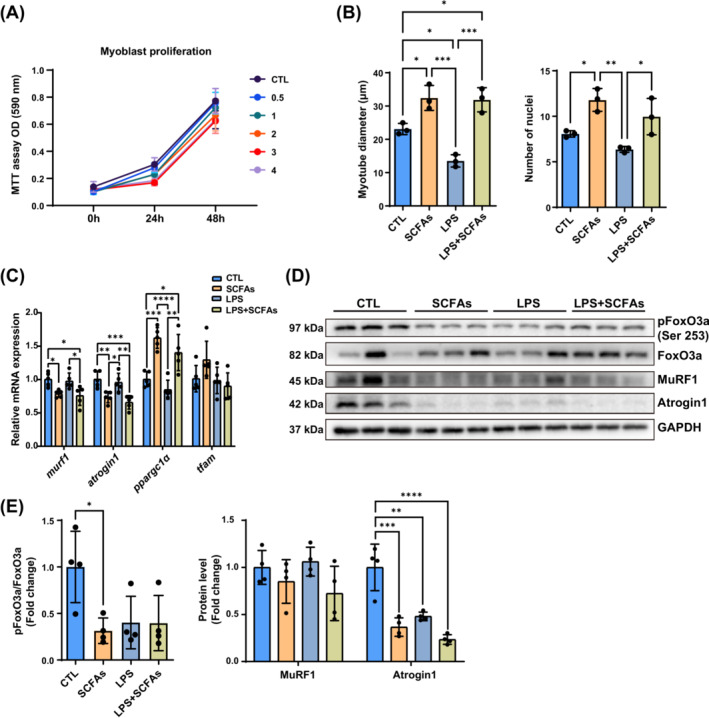
The proliferation of C2C12 myoblast, myotube structure, mRNA, and protein expression with or without SCFAs and LPS. (A) Cell proliferation detection with different concentration of SCFAs (*n* = 3–5 holes per group). Concentration 1 as 0.6 mM acetate, 0.25 mM butyrate, 0.15 mM propionate. 0.5, 2, 3, 4 represent the fold of concentration 1. CTL: black; 0.5: blue; 1: green; 2: orange; 3: red; 4: purple. (B) Average myotube diameters and nuclei number amongst groups (*n* = 3 plates of cells per group, 8–10 myofibre per plate). (C) The changes of mRNA expression of atrophy and mitochondria biogenesis genes after SCFAs without and with LPS (*n* = 5). (D, E) The activation of FoxO3a signalling pathways amongst groups (*n* = 4). *P < 0.05, **P < 0.01, ***P < 0.001, ****P < 0.0001, by two‐way ANOVA with Šídák's multiple comparison test.

In vitro, we used different concentration of SCFAs cocktail (60:25:15) to investigate the effect of SCFAs on C2C12 myoblast proliferation. There was a trend of reduced cell number when increased the concentration of SCFAs cocktail from 0.6, 0.25, 0.15 mM to higher folds. We did not find marked differences of cell proliferation or toxicity amongst SCFAs‐treated myoblasts compared with the control group (Figure 7A). Since P8 mice had higher serum concentration of LPS, we simulated the in vivo environment by adding LPS into the cell culture medium. Similar to in vivo studies, LPS‐treated cells had declined myotube diameters compared with the control group mainly due to pro‐inflammatory status (Figure S2C). SCFAs‐treated myotubes with or without LPS had larger diameters compared with the control and LPS groups. Additionally, SCFAs treatment also increased the number of nuclei of myotubes (Figure 7B). Reduced expression of muscle atrophic genes *atrogin1* and *murf1*, and increased mitochondria biogenesis gene *ppargc1α* were found in SCFAs‐treated groups compared with control groups with or without LPS (Figure 7C). At protein levels, decreased FoxO3a activation and Atrogin1 protein expression were found in the SCFA‐treated group without LPS. A trend of lower protein level of Atrogin1 (2‐fold lower) but not FoxO3a activation was found in the LPS + SCFAs group compared with the LPS group (Figure 7D,E).

### The suppression of SCFAs effects on myotubes by mTOR inhibitor

**Figure 8:**
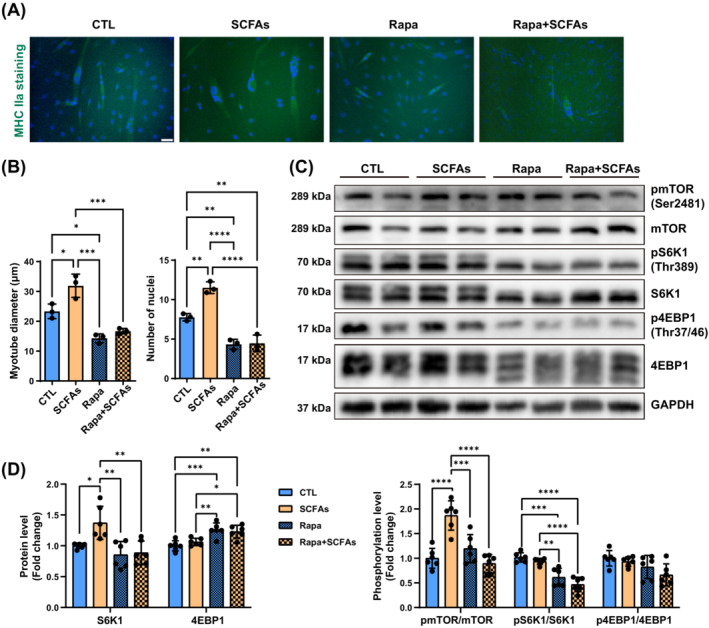
C2C12 myotube structure and protein expression with or without SCFAs and rapamycin. (A) MHC IIa staining of myotubes (scale bar = 50 μm). (B) Average myotube diameters and nuclei number amongst groups (*n* = 3 plates of cells per group, 8–10 myofibre per plate). (C, D) The activation of mTOR signalling pathways after SCFAs treatment with or without rapamycin (*n* = 6). *P < 0.05, **P < 0.01, ***P < 0.001, ****P < 0.0001, by two‐way ANOVA with Šídák's multiple comparison test.

In vivo studies showed that SCFAs potentially improved muscle status through mTOR signalling pathways. After the administration of rapamycin, an inhibitor of mTORC1, cell death rate was significantly increased, and the improvement of myotube diameters and nuclei number disappeared (Figure 8A,B). SCFAs treatment increased the mTOR activation and the protein level of S6K1 compared with the control group. The Rapa + SCFAs group had lower protein level of pS6K1 (Figure S2D), higher protein level of 4EBP1, and lower activation of S6K1 and mTOR compared with the SCFAs group. However, no significant increment of S6K1 and 4EBP1 phosphorylation after SCFAs treatment (Figure 8C,D). Under the LPS condition, similar results were found in myotube structures (Figure S3A). Higher activation of mTOR and S6K1 protein level were also found in the LPS + SCFAs group compared with the LPS group, but not in the LPS + Rapa + SCFAs group (Figure S3B). CT values of housekeeping gene were shown in Figure S4.

## Discussion

Sarcopenia is a prevalent muscle disorder in old people, and novel treatments are warranted. It is known that old people with sarcopenia have lower levels of SCFAs, and the supplement could potentially improve muscle health.^12^, ^27^ However, single acetate or butyrate treatment showed mild positive effects on muscle in aged animal models, and mechanisms are still unclear.^16^, ^17^ As reported by previous studies, the combination of the main components of SCFAs which is similar to the physiological concentration, may be more useful for sarcopenia.^18^ 10‐month‐old SAMP8 can be regarded as sarcopenic according to their lower muscle mass and function compared with the age‐matched SAMR1.^20^ In our study, sarcopenic SAMP8 had different gut microbiota composition compared with non‐sarcopenic SAMR1 according to beta diversity. We showed that SAMP8 had higher abundance of family Lachnospiraceae, but lower abundance of family Lactobacillaceae that is related to healthy aging.^9^ Although Lachnospiraceae family contained potential producers of SCFAs, SAMR1 had higher circulatory butyric acid than SAMP8 by GC–MS detection. After the SCFAs cocktail treatment, SAMP8 showed higher propionic acid than control SAMP8, and reached to a similar concentration of butyric acid compared with SAMR1. It indicated exogenous SCFAs administration could improve SCFAs levels in the circulation. The intestinal barrier function was impaired in the sarcopenic SAMP8, reflecting by higher serum LPS concentration, and lower protein expression of Muc2, Occludin, and Claudin1. After the SCFAs treatment, decreased LPS levels and higher Muc2 and Claudin1 were found, but Occludin and E‐cadherin protein were not affected. SCFAs could enhance the intestinal epithelial barrier function in various disease, such as IBD and metabolic disorders.^28^, ^29^ Fewer pro‐inflammatory cells and lower *il‐1β* expression were found in the colon of SCFAs treated mice. Therefore, SCFAs not only protected the gut barrier, enhanced circulatory SCFAs levels, but also attenuated inflammation status of the colon.

With regard to muscle, SCFAs improved muscle mass, strength, endurance, muscular glycogen, and myofibre CSA. TA mass significantly increased after the treatment. GAS myofibre CSA also improved by SCFAs, even it was still lower than SAMR1. SCFAs‐treated mice had lower mRNA expression of *murf1* and *atrogin1*, which were two muscle atrophy markers. The type I muscle fibre was increased in sarcopenic mice, but not reduced after the SCFAs treatment. From transcriptomic analysis, it may be due to the increment of both *rcan1* and *nfatc1*, which reached to a balance of muscle fibre switch. NFATc1 is related to fast‐to‐slow muscle fibre switching, and Rcan1 is an endogenous inhibitor of NFATc1 activation.^30^ Previous studies showed that acetic acid could proliferate slow twitch fibres through the improvement of intracellular calcium influx.^31^ The single butyrate administration did not change the fibre type significantly.^16^ We found a higher percentage of type IIb fibre (fast twitch) which may contribute to increased GAS muscle fibre CSA and ex vivo function after SCFAs treatment. Furthermore, treated mice not only had improved muscle function including increased grip strength, tetanic and twitch force, but also had better anti‐fatigue capacity. The increased mitochondria function including improved expression of PGC1α, as well as the activation of AMPK signalling pathways may increase in anti‐fatigue capacity of slow fibre.^17^ Besides, we also detected higher glycogen content in muscle of SCFAs‐treated mice which could produce energy for prolonged physical activities.^14^

Based on RNA‐seq findings, although chemokine increments were mainly associated with muscle dysfunction in SAMP8 compared with SAMR1, SCFAs did not benefit muscle inflammation status in our study. Nevertheless, SCFAs‐activated pathways have also showed potential in muscle improvement, including AMPK, insulin, and mTOR signalling pathways.^14^, ^32^ We first explored the changes of insulin‐like growth factors and related pathways, and observed markedly enhanced expression of *igf1*, *igf1r*, and *igfbp3* in the treated group. Previous studies reported insulin is a key regulator of glycogenesis, which may be a mechanism linking SCFAs and higher glycogen content in muscle.^14^ To further detect the AKT/mTOR pathways which might be activated by IGF1, we measured the activation of AKT, mTOR and its downstream proteins. Improved AKT/mTOR activation, and higher protein expression of S6K1 as well as slight inhibition of 4EBP1 activation might contribute to the muscle protein synthesis in sarcopenic mice after SCFAs treatment. Therefore, the activation of AKT/mTOR might be attributed to SCFAs‐induced changes of IGF status in sarcopenia. Intramuscular fat and mitochondrial dysfunction are also pathogenic factors of sarcopenia.^6^, ^33^ SCFAs not only improved the lipid metabolism, but also the mitochondrial biogenesis. Similar to aerobic exercise, SCFAs activated AMPK pathways and PGC1α expression.^34^ The expression of *sirt1*, a gene related to mitochondrial biogenesis, also increased after the treatment. The increased mtDNA/nuDNA indicated more mitochondria in the muscle. PPARδ could reduce the intramuscular lipid in muscle by improving lipid oxidation.^35^ SCFAs may improve lipid metabolism in muscle through higher expression of *pparδ*.

In vitro, SCFAs did not improve the proliferation of C2C12 myoblasts, but also no obvious toxicity was observed when the concentration was changed. We previously detected that LPS significantly elevated in sarcopenic mice. In C2C12 myotubes, we found LPS shrunk the diameters in myotubes, and SCFAs could reverse the adverse changes. SCFAs down‐regulated the expression of *murf1* and *agrogin1* at mRNA levels, and significantly reduced protein levels of Atrogin1 through the inhibition of FoxO3a phosphorylation. Although the reduction of Atrogin1 protein in the SCFAs+LPS group was not as obvious as in the SCFAs group when compared with their control groups, SCFAs could still decrease the mRNA expression of *murf1* and *atrogin1*, as well as improved *ppargc1α* when added LPS. The positive effect of SCFAs on myotubes were established with or without LPS.^15^, ^18^ Despite this, the reduction of circulating LPS concentration via improved gut barrier function after SCFA treatment may be the primary benefit for muscle growth. Considering the AKT/mTOR signalling pathways, we blocked the downstream mTOR activation by rapamycin,^36^ and found remarkable cell apoptosis in two rapamycin groups with or without SCFAs. Rapamycin groups had shorter myotube diameter length and fewer nuclei, indicating the decline of differentiation and fusion capacity of myocytes. SCFAs improved the mTOR activation and S6K1 expression as in vivo, but not altered phosphorylation levels of neither S6K1 nor 4EBP1 compared with the CTL group. After the inhibition of mTOR, the improvement of myotubes disappeared in Rapa + SCFAs group. The activation of mTOR and S6K1, protein expression of S6K1 were lower, whilst protein expression of 4EBP1 was higher in Rapa + SCFAs group than SCFAs group. Previous studies reported that butyrate could activate mTOR pathway of LPS and high glucose‐induced myoblasts.^15^ SCFAs also activated mTOR and S6K1 protein level in C2C12 with LPS in our studies, which could be inhibited by rapamycin. It indicated that mTOR signalling pathway is important in the effect of SCFAs on muscle.

This is the first study reporting that SCFAs cocktail mainly produced by beneficial gut bacteria could attenuate age‐related sarcopenia through the mTOR signalling pathways. Energy and lipid metabolisms, as well as mitochondria biogenesis were also involved. There are several limitations of this study. Firstly, only male mice and cells were used to detect the effect of SCFAs cocktail, but the effect may be different in female mice and human. The finding of in vitro study should be further validated in vivo. Single dose or duration was detected at this time, and further study should focus on the most efficiency treatment strategy. Clinical translation of SCFAs for old people with sarcopenia is warranted.

In conclusion, aged mice with or without sarcopenia had different gut microbiota composition, gut permeability and inflammation, as well as circulatory SCFAs concentration. The cocktail of SCFAs could promote tibialis anterior mass, grip strength, gastrocnemius ex vivo function, anti‐fatigue capacity, and colon health of sarcopenic mice. Increased myofibre cross‐sectional area, proportion of type IIb fibre, improved lipid metabolism and mitochondria biogenesis were found after the treatment. AKT/mTOR/S6K1 and AMPK/PGC1α signalling pathways were involved in the beneficial role of SCFAs in muscle. The inhibition of mTOR pathways attenuated the positive effect of SCFAs on myotube growth. SCFAs cocktail with the physiological concentration ratio can potentially be therapeutic strategies for old people with sarcopenia.

## Conflict of interest

The authors declare that they have no competing interests.

## Funding

This study was supported by grants from the General Research Fund Early Career Scheme, HKSAR Research Grant Council (24108519), Research Grants Council (RGC) General Research Fund (14116223), and the Collaborative Research Fund (C4032‐21GF).

## Online supplementary material

16S rDNA sequencing data of gut microbiota, and muscle RNA‐seq data are accessible under PRJNA1059771. Additional supporting information may be found online in the Supporting Information section at the end of the article.

## Supporting information


**Figure S1.** Calculation method of area percentage of GAS intramuscular lipids. Oil Red O staining (AAPR101, PythonBio) was performed to detect lipid content in muscle sections. Images were analysed using ImageJ software. The analysis was started by importing the RGB images into the ImageJ software, then splitting the channels with the commands: *Image > Colour > Split Channels*. The blue channel of the 8‐bit grey scale images with clear background were used for lipid content calculation. Threshold was set with commands: *Image > Adjust > Threshold*, default setting was used with slight adjustment according to the images. The area of lipid droplets was measured with commands: *Analyse > Analyse particle*, with following settings, *size: 0‐Infinity; Circularity: 0.00–1.00; boxes of Display results, Clear results, Summarize* and *Exclude on edges* were checked. From the summary table, %Area was obtained, which represented the area occupied by lipid droplets (%) calculated as the ratio of area of lipid droplets to that of background automatically.
**Figure S2.** Heart staining, TA muscle protein expression, cytokines mRNA expression of C2C12 myotubes with LPS, and phosphorylated protein levels of C2C12 treated with or without SCFAs and rapamycin. (A) Sirus red and H&E staining showed no significant pathological changes between mice with sodium chloride and SCFAs (CTL R1, CTL P8, SCFAs P8) compared to untreated mice (R1, P8). (B) Protein levels of pS6K1 and p4EBP1 in TA muscle amongst groups (*n* = 6). (C) Pro‐inflammatory cytokines mRNA expression of C2C12 myotubes after LPS treatment (*n* = 5). (D) Protein levels of pS6K1 and p4EBP1 in C2C12 treated with or without SCFAs and rapamycin (*n* = 6). * *P* < 0.05, ** *P* < 0.01, *** *P* < 0.001, **** *P* < 0.0001, by one‐ way ANOVA with Tukey's analysis, Student's unpaired t‐test, or two‐way ANOVA with Šídák's multiple comparison test.
**Figure S3.** LPS‐induced C2C12 myotube structure and protein levels with or without SCFAs and rapamycin. (A) MHC IIa staining of myotubes (scale bar = 50 μm), and average myotube diameters and nuclei number amongst groups (*n* = 3 plates of cells per group, calculation of 8–10 myotubes per plate). (B) Protein expression in mTOR signalling pathways after SCFAs treatment with or without rapamycin in LPS‐induced C2C12 myotubes (*n* = 6). * *P* < 0.05, ** *P* < 0.01, *** *P* < 0.001, **** *P* < 0.0001, by two‐way ANOVA with Šídák's multiple comparison test.
**Figure S4.** CT value of housekeeping genes for all RT‐qPCR experiments. One‐way ANOVA with Tukey's analysis, two‐way ANOVA with Šídák's multiple comparison test, and Student's unpaired t‐test were performed.
**Table S1.** Primer sequence for PCR.
**Table S2.** Abbreviations list.

## References

[1] Chen LK , Woo J , Assantachai P , Auyeung TW , Chou MY , Iijima K , et al. Asian Working Group for Sarcopenia: 2019 Consensus Update on Sarcopenia Diagnosis and Treatment. J Am Med Dir Assoc 2020;21:300–307. 32033882 10.1016/j.jamda.2019.12.012

[2] Petermann‐Rocha F , Balntzi V , Gray SR , Lara J , Ho FK , Pell JP , et al. Global prevalence of sarcopenia and severe sarcopenia: a systematic review and meta‐analysis. J Cachexia Sarcopenia Muscle 2021;13:86–99. 34816624 10.1002/jcsm.12783 PMC8818604

[3] Cheng KY , Chow SK , Hung VW , Wong CH , Wong RM , Tsang CS , et al. Diagnosis of sarcopenia by evaluating skeletal muscle mass by adjusted bioimpedance analysis validated with dual‐energy X‐ray absorptiometry. J Cachexia Sarcopenia Muscle 2021;12:2163–2173. 34609065 10.1002/jcsm.12825 PMC8718029

[4] Wong RMY , Ho WT , Wai LS , Li W , Chau WW , Chow KS , et al. Fragility fractures and imminent fracture risk in Hong Kong: one of the cities with longest life expectancies. Arch Osteoporos 2019;14:104. 31659457 10.1007/s11657-019-0648-4

[5] Zhang X , Zhang W , Wang C , Tao W , Dou Q , Yang Y . Sarcopenia as a predictor of hospitalization among older people: a systematic review and meta‐analysis. BMC Geriatr 2018;18:188. 30134867 10.1186/s12877-018-0878-0 PMC6103964

[6] Liu C , Wong PY , Chung YL , Chow SK , Cheung WH , Law SW , et al. Deciphering the “obesity paradox” in the elderly: A systematic review and meta‐analysis of sarcopenic obesity. Obes Rev 2022;24:e13534. 36443946 10.1111/obr.13534

[7] Cruz‐Jentoft AJ , Bahat G , Bauer J , Boirie Y , Bruyere O , Cederholm T , et al. Sarcopenia: revised European consensus on definition and diagnosis. Age Ageing 2019;48:16–31. 30312372 10.1093/ageing/afy169 PMC6322506

[8] Cheng KY , Bao Z , Long Y , Liu C , Huang T , Cui C , et al. Sarcopenia and Ageing. Subcell Biochem 2023;103:95–120. 37120466 10.1007/978-3-031-26576-1_6

[9] Ragonnaud E , Biragyn A . Gut microbiota as the key controllers of "healthy" aging of elderly people. Immunity Ageing: IA 2021;18:2. 10.1186/s12979-020-00213-w PMC7784378 33397404

[10] van der Hee B , Wells JM . Microbial Regulation of Host Physiology by Short‐chain Fatty Acids. Trends Microbiol 2021;29:700–712. 33674141 10.1016/j.tim.2021.02.001

[11] Liu C , Cheung WH , Li J , Chow SK , Yu J , Wong SH , et al. Understanding the gut microbiota and sarcopenia: a systematic review. J Cachexia Sarcopenia Muscle 2021;12:1393–1407. 34523250 10.1002/jcsm.12784 PMC8718038

[12] Guan L , Cao Z , Pan Z , Zhao C , Xue M , Yang F , et al. Butyrate promotes C2C12 myoblast proliferation by activating ERK/MAPK pathway. Mol Omics 2023;19:552–559. 37204279 10.1039/d2mo00256f

[13] Li C , Li Y , Wang N , Ge Z , Shi Z , Wang J , et al. Intestinal Permeability Associated with the Loss of Skeletal Muscle Strength in Middle‐Aged and Older Adults in Rural Area of Beijing, China. Healthcare (Basel) 2022;10. 10.3390/healthcare10061100 PMC9223217 35742149

[14] Frampton J , Murphy KG , Frost G , Chambers ES . Short‐chain fatty acids as potential regulators of skeletal muscle metabolism and function. Nat Metab 2020;2:840–848. 32694821 10.1038/s42255-020-0188-7

[15] Tang G , Du Y , Guan H , Jia J , Zhu N , Shi Y , et al. Butyrate ameliorate skeletal muscle atrophy in Diabetic Nephropathy via enhancing gut barrier function and FFA2‐mediated PI3K/AKT/mTOR signals. Br J Pharmacol 2021. 10.1111/bph.15693 34638162

[16] Walsh ME , Bhattacharya A , Sataranatarajan K , Qaisar R , Sloane L , Rahman MM , et al. The histone deacetylase inhibitor butyrate improves metabolism and reduces muscle atrophy during aging. Aging Cell 2015;14:957–970. 26290460 10.1111/acel.12387 PMC4693467

[17] Maruta H , Abe R , Yamashita H . Effect of Long‐Term Supplementation with Acetic Acid on the Skeletal Muscle of Aging Sprague Dawley Rats. Int J Mol Sci 2022;23. 35563082 10.3390/ijms23094691 PMC9101554

[18] Lahiri S , Kim H , Garcia‐Perez I , Reza MM , Martin KA , Kundu P , et al. The gut microbiota influences skeletal muscle mass and function in mice. Sci Transl Med 2019;11:11. 10.1126/scitranslmed.aan5662 PMC7501733 31341063

[19] Seethaler B , Nguyen NK , Basrai M , Kiechle M , Walter J , Delzenne NM , et al. Short‐chain fatty acids are key mediators of the favorable effects of the Mediterranean diet on intestinal barrier integrity: data from the randomized controlled LIBRE trial. Am J Clin Nutr 2022;116:928–942. 36055959 10.1093/ajcn/nqac175

[20] Guo AY , Leung KS , Siu PM , Qin JH , Chow SK , Qin L , et al. Muscle mass, structural and functional investigations of senescence‐accelerated mouse P8 (SAMP8). Exp Anim 2015;64:425–433. 26193895 10.1538/expanim.15-0025 PMC4637380

[21] Xie WQ , He M , Yu DJ , Wu YX , Wang XH , Lv S , et al. Mouse models of sarcopenia: classification and evaluation. J Cachexia Sarcopenia Muscle 2021;12:538–554. 33951340 10.1002/jcsm.12709 PMC8200444

[22] Wang J , Cui C , Chim YN , Yao H , Shi L , Xu J , et al. Vibration and beta‐hydroxy‐beta‐methylbutyrate treatment suppresses intramuscular fat infiltration and adipogenic differentiation in sarcopenic mice. J Cachexia Sarcopenia Muscle 2020;11:564–577. 31994349 10.1002/jcsm.12535 PMC7113529

[23] Klein AB , Nicolaisen TS , Ortenblad N , Gejl KD , Jensen R , Fritzen AM , et al. Pharmacological but not physiological GDF15 suppresses feeding and the motivation to exercise. Nat Commun 2021;12:1041. 33589633 10.1038/s41467-021-21309-x PMC7884842

[24] Lipinska P , Pawlak P , Warzych E . Species and embryo genome origin affect lipid droplets in preimplantation embryos. Front Cell Dev Biol 2023;11. 10.3389/fcell.2023.1187832 PMC10217358 37250899

[25] Mohr AE , Crawford M , Jasbi P , Fessler S , Sweazea KL . Lipopolysaccharide and the gut microbiota: considering structural variation. FEBS Lett 2022;596:849–875. 35262962 10.1002/1873-3468.14328

[26] Cao YY , Wang Z , Yu T , Zhang Y , Wang ZH , Lu ZM , et al. Sepsis induces muscle atrophy by inhibiting proliferation and promoting apoptosis via PLK1‐AKT signalling. J Cell Mol Med 2021;25:9724–9739. 34514712 10.1111/jcmm.16921 PMC8505846

[27] Huang T , Liu C , Cui C , Zhang N , Cheung WH , Wong RMY . Potential of Fatty Acids in Treating Sarcopenia: A Systematic Review. Nutrients 2023;15:15. 10.3390/nu15163613 PMC10459935 37630803

[28] You H , Tan Y , Yu D , Qiu S , Bai Y , He J , et al. The Therapeutic Effect of SCFA‐Mediated Regulation of the Intestinal Environment on Obesity. Front Nutr 2022;9:886902. 35662937 10.3389/fnut.2022.886902 PMC9157426

[29] Parada Venegas D , De la Fuente MK , Landskron G , Gonzalez MJ , Quera R , Dijkstra G , et al. Short Chain Fatty Acids (SCFAs)‐Mediated Gut Epithelial and Immune Regulation and Its Relevance for Inflammatory Bowel Diseases. Front Immunol 2019;10:277. 30915065 10.3389/fimmu.2019.00277 PMC6421268

[30] Ehlers ML , Celona B , Black BL . NFATc1 controls skeletal muscle fiber type and is a negative regulator of MyoD activity. Cell Rep 2014;8:1639–1648. 25242327 10.1016/j.celrep.2014.08.035 PMC4180018

[31] Maruta H , Yamashita H . Acetic acid stimulates G‐protein‐coupled receptor GPR43 and induces intracellular calcium influx in L6 myotube cells. PLoS ONE 2020;15:e0239428. 32997697 10.1371/journal.pone.0239428 PMC7526932

[32] Bodine SC , Stitt TN , Gonzalez M , Kline WO , Stover GL , Bauerlein R , et al. Akt/mTOR pathway is a crucial regulator of skeletal muscle hypertrophy and can prevent muscle atrophy in vivo. Nat Cell Biol 2001;3:1014–1019. 11715023 10.1038/ncb1101-1014

[33] Liu C , Cheng KY‐K , Tong X , Cheung W‐H , Chow SK‐H , Law SW , et al. The role of obesity in sarcopenia and the optimal body composition to prevent against sarcopenia and obesity. Front Endocrinol 2023;14:14. 10.3389/fendo.2023.1077255 PMC10016224 36936175

[34] Liang J , Zhang H , Zeng Z , Wu L , Zhang Y , Guo Y , et al. Lifelong Aerobic Exercise Alleviates Sarcopenia by Activating Autophagy and Inhibiting Protein Degradation via the AMPK/PGC‐1alpha Signaling Pathway. Metabolites 2021;11:11. 10.3390/metabo11050323 PMC8157243 34069829

[35] de Lange P , Lombardi A , Silvestri E , Goglia F , Lanni A , Moreno M . Peroxisome Proliferator‐Activated Receptor Delta: A Conserved Director of Lipid Homeostasis through Regulation of the Oxidative Capacity of Muscle. PPAR Res 2008;2008:172676. 18815630 10.1155/2008/172676 PMC2547483

[36] Ballou LM , Lin RZ . Rapamycin and mTOR kinase inhibitors. J Chem Biol 2008;1:27–36. 19568796 10.1007/s12154-008-0003-5 PMC2698317

[37] von Haehling S , Coats AJS , Anker SD . Ethical guidelines for publishing in the Journal of Cachexia, Sarcopenia and Muscle: Update 2023. J Cachexia Sarcopenia Muscle 2023;14:2981–2983. 38148513 10.1002/jcsm.13420 PMC10751405

