# Supplementary material for: Short‐chain fatty acids enhance muscle mass and function through the activation of mTOR signalling pathways in sarcopenic mice
Source: J Cachexia Sarcopenia Muscle. 2024 Oct 31;15(6):2387–401. doi: 10.1002/jcsm.13573 (PMC11634463; doi:10.1002/jcsm.13573)
Supplement: Supplementary file 1 — Figure S1. Calculation method of area percentage of GAS intramuscular lipids. Oil Red O staining (AAPR101, PythonBio) was performed to detect lipid content in muscle sections. Images were analysed using ImageJ software. The analysis was started by importing the RGB images into the ImageJ software, then splitting the channels with the commands: Image > Colour > Split Channels. The blue channel of the 8‐bit grey scale images with clear background were used for lipid content calculation. Threshold was set with commands: Image > Adjust > Threshold, default setting was used with slight adjustment according to the images. The area of lipid droplets was measured with commands: Analyse > Analyse particle, with following settings, size: 0‐Infinity; Circularity: 0.00–1.00; boxes of Display results, Clear results, Summarize and Exclude on edges were checked. From the summary table, %Area was obtained, which represented the area occupied by lipid droplets (%) calculated as the ratio of area of lipid droplets to that of background automatically. Figure S2. Heart staining, TA muscle protein expression, cytokines mRNA expression of C2C12 myotubes with LPS, and phosphorylated protein levels of C2C12 treated with or without SCFAs and rapamycin. (A) Sirus red and H&E staining showed no significant pathological changes between mice with sodium chloride and SCFAs (CTL R1, CTL P8, SCFAs P8) compared to untreated mice (R1, P8). (B) Protein levels of pS6K1 and p4EBP1 in TA muscle amongst groups (n = 6). (C) Pro‐inflammatory cytokines mRNA expression of C2C12 myotubes after LPS treatment (n = 5). (D) Protein levels of pS6K1 and p4EBP1 in C2C12 treated with or without SCFAs and rapamycin (n = 6). * P < 0.05, ** P < 0.01, *** P < 0.001, **** P < 0.0001, by one‐ way ANOVA with Tukey's analysis, Student's unpaired t‐test, or two‐way ANOVA with Šídák's multiple comparison test. Figure S3. LPS‐induced C2C12 myotube structure and protein levels with or without SCFAs and rapamycin. (A) MHC IIa sta [file JCSM-15-2387-s001.pdf]

Supporting Information

Title: Short-chain fatty acids enhance muscle mass and function through the activation of mTOR signaling pathways in sarcopenic mice

Material and methods

Stool sample DNA extraction and 16S rDNA sequencing

Mice were placed into a sterile box individually for fresh stool collection in the morning. For each mouse, 2–3 fresh fecal pellets were collected using sterile forceps and put into an autoclaved tube for preventing environmental contamination of stool samples. After collection, the tubes were frozen with liquid nitrogen instantly, and then translocated to -80 °C freezer for storage. The genomic DNA in mouse stool samples was extracted using the E.Z.N.A. ®Stool DNA Kit (D4015, Omega, Inc., USA) according to manufacturer's instructions. The total extracted DNA was diluted in 50 μL of Elution buffer. The hypervariable region of the 16S rDNA V4–V5 was amplified using forward 5'-GTGCCAGCMGCCGCGG-3', and reverse 5'-CCGTCAATTCMTTTRAGTTT-3' primers. PCR products were purified via AMPure XT beads (Beckman Coulter Genomics, Danvers, MA, USA), and quantified by Qubit (Invitrogen, USA). The size of amplicon library was assessed by Agilent 2100 Bioanalyzer (Agilent, USA), and the quantity was evaluated by Library Quantification Kit for Illumina (Kapa Biosciences, Woburn, MA, USA). The 16S rDNA sequencing was performed on the NovaSeq PE250 platform (Illumina, San Diego, USA).

Gas chromatography-mass spectrometry (GC-MS) analysis of serum SCFAs

Blood samples were drawn from mice and centrifuged at 4,000 rpm for 10 min. The serum was then extracted and stored in -80 °C freezer until use. For each 100 μL serum sample from the mice, 0.05 mL 50% H_2SO_4 and 0.2 mL 2-Methylvaleric acid were

added as internal standard. After 30 s vortex and 10 min oscillation, samples were ultrasonicated for 10 min in ice water and then centrifuged at 10,000 rpm for 15 min at 4 °C. Samples were kept at -20 °C for 30 min, and the supernatants were used for GC-MS analysis. GC2030-QP2020 NX GC-MS (SHIMADZU, Japan) with a HP-FFAP capillary column were used. In split mode (5:1), 1 μL of the analyte was injected using helium as the carrier gas, with 3 mL min^{-1} front inlet purge flow and 1 mL min^{-1} gas flow rate through the column. The initial temperature was maintained at 80 °C for 1 min and then increased to 200 °C at a rate of 10 °C min^{-1} for 5 min. Subsequently, it was kept at 240 °C for 1 min at a rate of 40 °C min^{-1}. The ion source, quad, injection, and transfer line temperatures were set at 200 °C, 150 °C, 240 °C, and 240 °C, respectively. Electron impact mode at the energy of -70 eV was used, while the mass spectrometry data were collected in Scan/SIM mode with a m/z range of 33–150 following a delayed solvent of 3.5 min.

RNA extraction and real-time PCR

RNA from EDL muscle, colon, and C2C12 myotubes were extracted with RNAiso plus (Takara, Japan). The RNA concentration was detected by NanoDrop 2000 (ND-2000; Thermo Scientific, USA). 500 ng RNA were reverse transcribed to cDNA by PrimeScript RT Reagent Kit (TaKaRa Biotechnology, Otsu, Japan). Reaction mixture of cDNA, primers and Power SYBR Green PCR Master Mix (Thermo Scientific, MA, USA) were added to a 384-well plate with 10 μL per well for quantitative real-time PCR on the Technology QuantStudio 12K Flex qPCR System (Thermo Scientific, Waltham, USA). Relative expression of candidate genes was analyzed with $2^{-\Delta\Delta Ct}$ method based on *gapdh* expression.

Western blot

Samples of TA, colon, and C2C12 myotubes were lysed in radioimmunoprecipitation assay (RIPA buffer) with protease/phosphatase inhibitor cocktail (CST, USA). The lysate was put on ice for 1h to fully extract tissue proteins or 30 min for cell protein extraction. After 15 min centrifugation at 15,000 rpm, the soluble protein was collected and quantified by Pierce BCA Protein Assay Kit (Thermo Scientific, Massachusetts, USA). The extracted protein was transferred to polyvinylidene fluoride (PVDF) membranes through electrophoresis. The primary antibodies were rabbit anti-mouse antibodies (1:2000) which included anti-mTOR (A2445, Abclonal, USA), anti-phospho-mTOR (Ser2481) (#2974, CST, USA), anti-S6K1 (ab32359, Abcam, Cambridge, UK), anti-phospho-p70 S6 Kinase (Thr389) (#9205, CST, USA), anti-eIF4EBP1 (ab32024, Abcam, Cambridge, UK), anti-phospho-4E-BP1 (Thr37/46) (#2855, CST, USA), anti-FoxO3a (#2497, CST, USA), anti-phospho-FoxO3a (Ser253) (#9466, CST, USA), anti-AMPKa1/AMPKa2 (A12718, Abclonal, USA) , anti-phospho-AMPKα (Thr172) (#2535, CST, USA), anti-PGC1α (A12348, Abclonal, USA), anti-Fbxo32 (known as Atrogin1) (A3699, Abclonal, USA), anti-Trim63 (known as Murf1) (A3101, Abclonal, USA), and E-cadherin (#3195, CST, USA). Antibodies anti-Muc2, anti-Claudin1, and anti-Occludin were same as staining used. Mouse IgG1 anti-GAPDH (MA5-15738, Invitrogen, USA) antibody (1:5000) was also used as internal control. Secondary antibodies (1:5000) were anti-rabbit IgG HRP-linked antibody (#7074, CST, USA), and goat anti-mouse IgG (H + L) antibody (ICN670281, Invitrogen, USA).

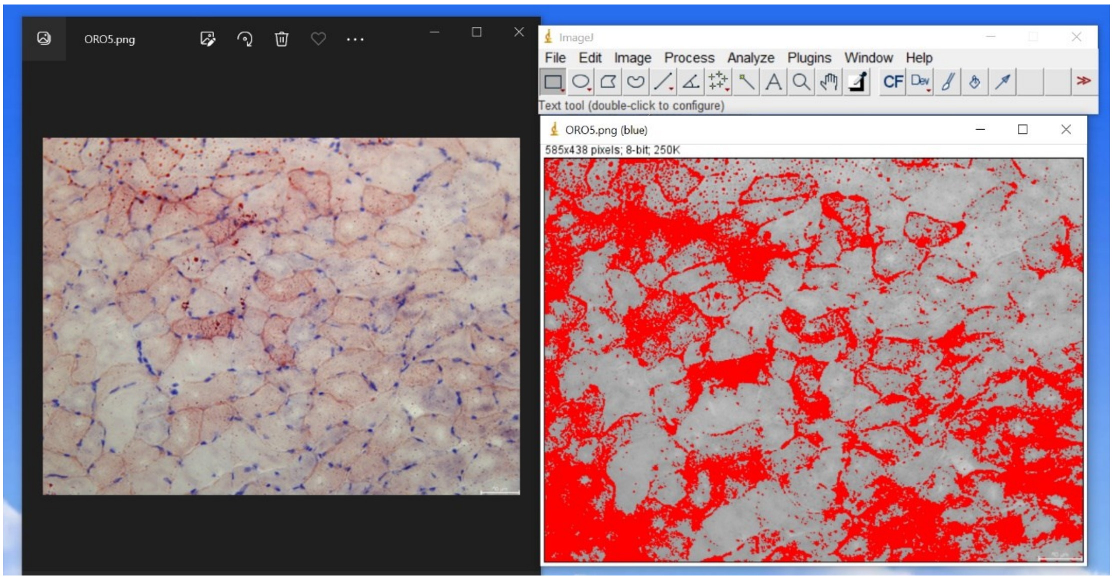

Figure S1 Calculation method of area percentage of GAS intramuscular lipids. Oil Red O staining (AAPR101, PythonBio) was performed to detect lipid content in muscle sections. Images were analyzed using ImageJ software. The analysis was started by importing the RGB images into the ImageJ software, then splitting the channels with the commands: *Image > Color > Split Channels*. The blue channel of the 8-bit grey scale images with clear background were used for lipid content calculation. Threshold was set with commands: *Image > Adjust > Threshold*, default setting was used with slight adjustment according to the images. The area of lipid droplets was measured with commands: *Analyze > Analyze particle*, with following settings, *size: 0-Infinity; Circularity: 0.00-1.00; boxes of Display results, Clear results, Summarize* and *Exclude on edges* were checked. From the summary table, %Area was obtained, which represented the area occupied by lipid droplets (%) calculated as the ratio of area of lipid droplets to that of background automatically.

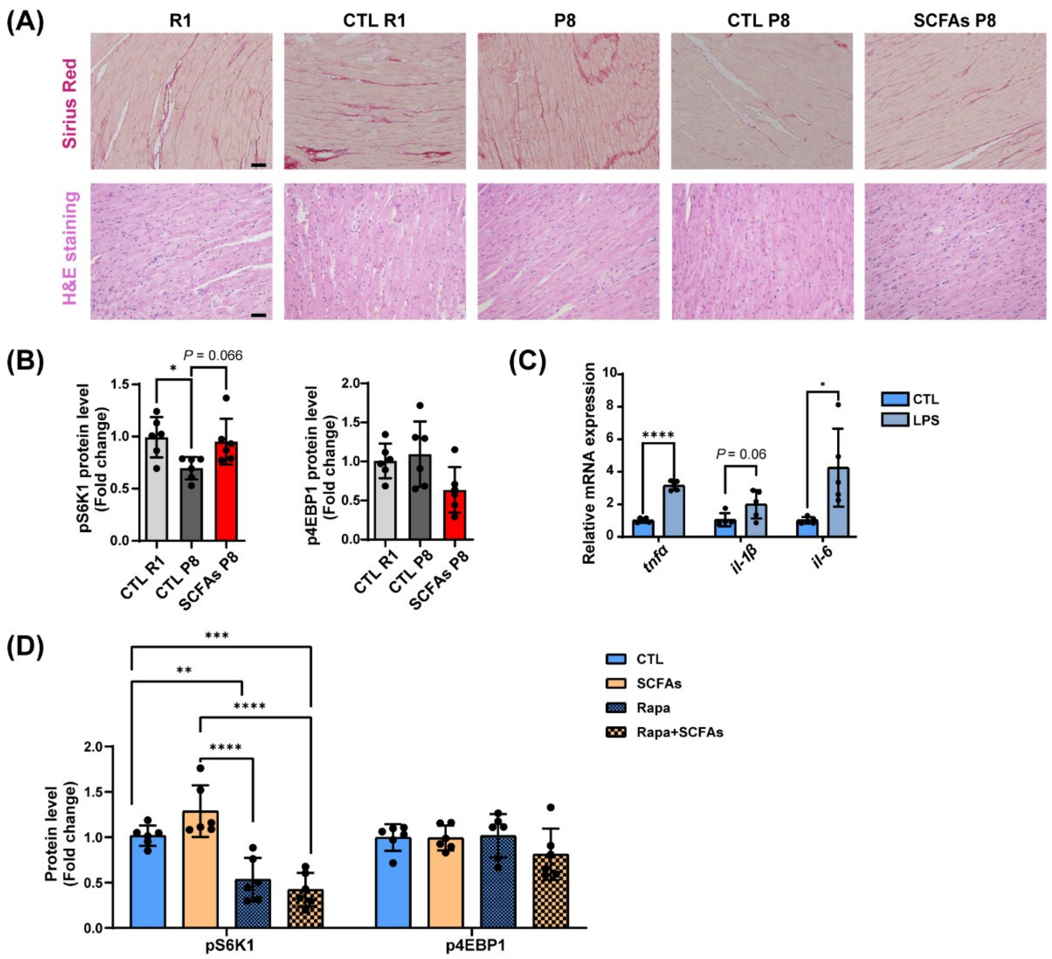

Figure S2 Heart staining, TA muscle protein expression, cytokines mRNA expression of C2C12 myotubes with LPS, and phosphorylated protein levels of C2C12 treated with or without SCFAs and rapamycin. (A) Sirus red and H&E staining showed no significant pathological changes between mice with sodium chloride and SCFAs (CTL R1, CTL P8, SCFAs P8) compared to untreated mice (R1, P8). (B) Protein levels of pS6K1 and p4EBP1 in TA muscle amongst groups (n = 6). (C) Pro-inflammatory cytokines mRNA expression of C2C12 myotubes after LPS treatment (n = 5). (D) Protein levels of pS6K1 and p4EBP1 in C2C12 treated with or without SCFAs and rapamycin (n = 6). * $P < 0.05$, ** $P < 0.01$, *** $P < 0.001$, **** $P < 0.0001$, by one-way ANOVA with Tukey's analysis, Student's unpaired t-test, or two-way ANOVA with Šídák's multiple comparison test.

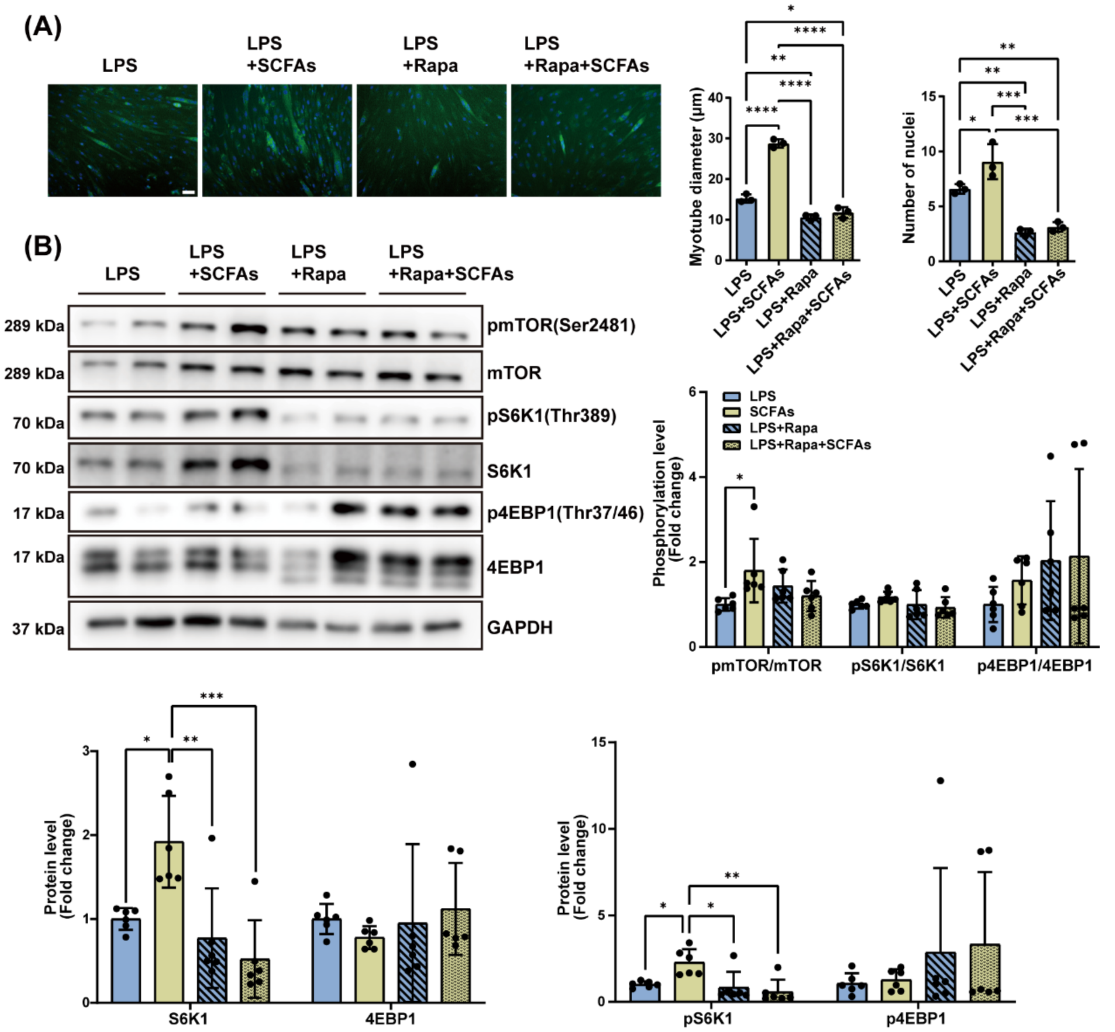

Figure S3 LPS-induced C2C12 myotube structure and protein levels with or without SCFAs and rapamycin. (A) MHC IIa staining of myotubes (scale bar = 50 μm), and average myotube diameters and nuclei number amongst groups (*n* = 3 plates of cells per group, calculation of 8-10 myotubes per plate). (B) Protein expression in mTOR signaling pathways after SCFAs treatment with or without rapamycin in LPS-induced C2C12 myotubes (*n* = 6). * $P < 0.05$, ** $P < 0.01$, *** $P < 0.001$, **** $P < 0.0001$, by two-way ANOVA with Šídák's multiple comparison test.

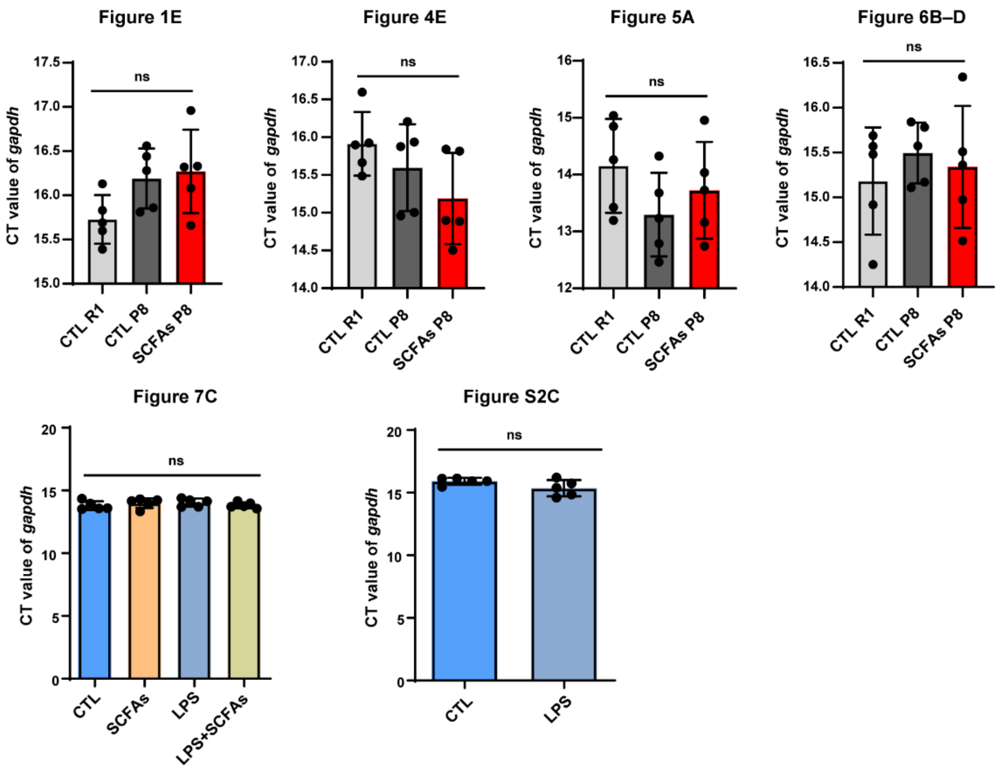

Figure S4 CT value of housekeeping genes for all RT-qPCR experiments. One-way ANOVA with Tukey's analysis, two-way ANOVA with Šídák's multiple comparison test, and Student's unpaired t-test were performed.

Table S1 Primer sequence for PCR

**Target Gene**	**Primer Sequence**
Atrogin1 Forward	CAGCTTCGTGAGCGACCTC
Atrogin1 Reverse	GGCAGTCGAGAAGTCCAGTC
C/EBPα Forward	CAAGAACAGCAACGAGTACCG
C/EBPα Reverse	GTCACTGGTCAACTCCAGCAC
GAPDH Forward	AACGACCCCTTCATTGAC
GAPDH Reverse	TCCACGACATACTCAGCAC
Igf1 Forward	AAATCAGCAGCCTTCCAACTC
Igf1 Reverse	GCACTTCCTCTACTTGTGTTCTT
Igf1r Forward	GTGGGGGCTCGTGTTTCTC
Igf1r Reverse	GATCACCGTGCAGTTTTCCA
Igfbp3 Forward	CCAGGAAACATCAGTGAGTCC
Igfbp3 Reverse	GGATGGAACTTGGAATCGGTCA
Igfbp5 Forward	CCCTGCGACGAGAAAGCTC
Igfbp5 Reverse	GCTCTTTTCGTTGAGGCAAACC
IL-1β Forward	GCAACTGTTCCTGAACTCAACT

IL-1β Reverse	ATCTTTTGGGGTCCGTCAACT
IL-6 Forward	TAGTCCTTCCTACCCCAATTTCC
IL-6 Reverse	TTGGTCCTTAGCCACTCCTTC
Murf1 Forward	GTGTGAGGTGCCTACTTGCTC
Murf1 Reverse	GCTCAGTCTTCTGTCCTTGGA
Myod1 Forward	CCACTCCGGGACATAGACTTG
Myod1 Reverse	AAAAGCGCAGGTCTGGTGAG
Myog Forward	GAGACATCCCCCTATTTCTACCA
Myog Reverse	GCTCAGTCCGCTCATAGCC
Nrf1 Forward	AGCACGGAGTGACCCAAAC
Nrf1 Reverse	TGTACGTGGCTACATGGACCT
Pgc1α Forward	TATGGAGTGACATAGAGTGTGCT
Pgc1α Reverse	CCACTTCAATCCACCCAGAAAG
PPARβ/δ Forward	TCCATCGTCAACAAAGACGGG
PPARβ/δ Reverse	ACTTGGGCTCAATGATGTCAC
Sirt1 Forward	GCTGACGACTTCGACGACG
Sirt1 Reverse	TCGGTCAACAGGAGGTTGTCT
Tfam Forward	ATTCCGAAGTGTTTTTCCAGCA
Tfam Reverse	TCTGAAAGTTTTGCATCTGGGT
TNFa Forward	CCCTCACACTCAGATCATCTTCT
TNFa Reverse	GCTACGACGTGGGCTACAG

Table S2 Abbreviations list

**Abbreviation**	**Full name**
AMPKα	AMP-activated protein kinase alpha
AWGS	Asian Working Group for Sarcopenia
C/EBPα	CCAAT/ enhancer binding protein alpha
cab39	Calcium-binding protein 39
clip1	CAP-Gly Domain Containing Linker Protein 1
CSA	cross-sectional area
DMEM	Dulbecco's Modified Eagle's medium
EDL	extensor digitorum longus
eIF4EBP1	Eukaryotic translation initiation factor 4E binding protein 1
ELISA	Enzyme linked immunosorbent assay
EWGSOP	European Working Group on Sarcopenia in Older People
Fbxo32/Atrogin1	F-box only protein 32
FDA	Food and Drug Administration

FDR	False Discovery Rate
FoxO3a	Forkhead box O3 a
GAPDH	Glyceraldehyde 3-phosphate dehydrogenase
GC-MS	Gas chromatography-mass spectrometry
GAS	gastrocnemius
HIF-1	Hypoxia-inducible factor-1
hk2	hexokinase 2
Igf1	Insulin-like growth factor-1
Igf1r	Insulin Like Growth Factor 1 Receptor
Igfbp3	Insulin-like growth factor-binding protein 3
Igfbp5	Insulin-like growth factor binding protein 3
IgG HRP-linked antibody	Immunoglobulin G Horseradish peroxidase-linked antibody
IL-1β	Interleukin-1 beta
IL-6	Interleukin 6
KEGG	Kyoto Encyclopedia of Genes and Genomes
LDA	Linear discriminant analysis
LEfSe	LDA effect size
LPS	lipopolysaccharide
MHC	myosin heavy chain
mknk2	MAP kinase-interacting serine/threonine-protein kinase 2
mtDNA	mitochondrial DNA
mTOR	Mammalian target of rapamycin
mTOR complex 1	mTORC1
Murf1	Muscle RING-finger protein-1
Myod1	Myogenic determination factor 1
Myog	Myogenin
nfatc1	Nuclear Factor of Activated T Cells 1
Nrf1	Nuclear respiratory factor 1
nuDNA	nuclear DNA
PCoA	Principal Coordinate Analysis
pfkfb3	6-Phosphofructo-2-Kinase/Fructose-2,6-Biphosphatase 3
PGC1α/ ppargc1α	Peroxisome proliferator-activated receptor gamma coactivator 1-alpha
PPARβ/δ	Peroxisome proliferator-activated receptor beta/delta
prkag2	Protein Kinase AMP-Activated Non-Catalytic Subunit Gamma 2

QUA	quadriceps
Rapa	rapamycin
rcan1	regulator of calcineurin 1
S6K1	Ribosomal protein S6 kinase beta-1
SAMP8/P8	senescent accelerated mouse prone 8
SAMR1/R1	senescence accelerated mouse resistant 1
SCFAs	short-chain fatty acids
Sirt1	Silent mating type information regulation 2 homologue 1
SOL	soleus
TA	tibialis anterior
Tfam	Transcription factor A, mitochondrial
TNFα	Tumor necrosis factor-alpha
Trim63	Tripartite motif containing 63
